# A Molecular Investigation of Malaria Infections From High-Transmission Areas of Southern Togo Reveals Different Species of *Plasmodium* Parasites

**DOI:** 10.3389/fmicb.2021.732923

**Published:** 2021-12-02

**Authors:** Kokouvi Kassegne, Si-Wei Fei, Koffigan Ananou, Kokou Sépénou Noussougnon, Komi Komi Koukoura, Eniola Michael Abe, Xiao-Kui Guo, Jun-Hu Chen, Xiao-Nong Zhou

**Affiliations:** ^1^School of Global Health, Chinese Center for Tropical Diseases Research, Shanghai Jiao Tong University School of Medicine, Shanghai, China; ^2^National Institute of Parasitic Diseases, Chinese Center for Diseases Control and Prevention (Chinese Center for Tropical Diseases Research), National Health Commission of the People’s Republic of China (NHC) Key Laboratory of Parasite and Vector Biology, World Health Organization (WHO) Collaborating Center for Tropical Diseases, National Center for International Research on Tropical Diseases, Shanghai, China; ^3^Centre Médico-Social Notre Dame de la Consolation, Atakpamé, Togo; ^4^Hôpital Bethesda d’Agou Nyogbo, Agou, Togo; ^5^Laboratoire des Sciences Biomédicales, Alimentaires et Santé Environnementale, Département des Analyses Biomédicales, Ecole Supérieure des Techniques Biologiques et Alimentaires, Université de Lomé, Lomé, Togo; ^6^Department of Social Work, Education and Community Wellbeing, Faculty of Health and Life Sciences, Northumbria University, Newcastle upon Tyne, United Kingdom

**Keywords:** malaria, molecular surveillance, *Plasmodium* species, phylogeny, Togo

## Abstract

Malaria particularly burdens people in poor and neglected settings across the tropics of Africa. Meanwhile, a large proportion of the Togo population have poor understanding of malaria epidemiology and parasites. This study carried out a molecular survey of malaria cases in southern Togo during 2017–2019. We estimated *Plasmodium* species infection rates and microscopic examination compliance with nested PCR results. Sensitivity and specificity analyses were performed in conjunction with predictive values. Also, phylogenetic characterization of species of malaria parasites was assessed. *Plasmodium* genus-specific nested PCR identified 565 positive cases including 536/611 (87.8%) confirmed cases from the microscopy-positive group and 29/199 (14.6%) diagnosed malaria cases from the microscopy-negative group. Our findings revealed a disease prevalence (69.8%) higher than that reported (25.5–55.1%) for the country. The diagnostic test had 94.9% sensitivity and 69.4% specificity, i.e., it missed 120 of the people who had malaria and about one-third of the people tested positive for the disease, which they did not have, respectively. In conjunction, the test showed 87.7% positive predictive value and 85.4% negative predictive value, which, from a clinical perspective, indicates the chance that a person with a positive diagnostic test truly has the disease and the probability that a person with a negative test does not have the disease, respectively. Further species-specific nested PCR followed by analysis of gene sequences confirmed species of malaria parasites and indicated infection rates for *Plasmodium falciparum* (Pf), 95.5% (540/565); *P. ovale* (Po), 0.5% (3/565); and *P. malariae* (Pm), 0.4% (2/565). In addition, 20 cases were coinfection cases of Pf-Po (15/565) and Pf-Pm (5/565). This study publicly reports, for the first time, a molecular survey of malaria cases in Togo and reveals the presence of other malaria parasites (Po and Pm) other than Pf. These findings might provide answers to some basic questions on the malaria scenario and, knowledge gained could help with intervention deployment for effective malaria control in Togo.

## Introduction

Malaria is a major debilitating infectious disease that affects millions of people and impedes public health and socioeconomic development in Africa (Kassegne et al., 2017). In tropical Africa, nearly 90% of deaths among young children are related to malaria (World Health Organization [WHO], 2018b).

Malaria transmission occurs throughout the year in Togo and is usually characterized by seasonal outbreaks with a peak during the rainy season. An estimated nearly 100% of the population of Togo lives in areas endemic for malaria—all the five administrative regions of the country having populations at risk of malaria—with disease prevalence rate estimated between 25.5 and 55.1% (World Health Organization [WHO], 2018a). In 2016, uncomplicated malaria was the first cause (41.7%) of outpatient consultations in public health centers and, hospitalization rate related to severe disease accounted for 20.5%, with 3.8% hospital mortality (Thomas et al., 2020). In Togo mortality rate associated with severe malaria is underestimated because most of the cases do not adhere to clinical consultations. For example, in 2017, > 60% of severe cases have not been to the hospital (Programe National de Lutte contre le Paludisme [PNLP], 2019). Children under 5 years old remain highly affected by malaria, with 35.4% uncomplicated outpatient cases, 58.4% hospitalized severe cases, and 69.7% deaths reported in 2017 (Programe National de Lutte contre le Paludisme [PNLP], 2019). Pregnant women accounted for 5% uncomplicated outpatients and 6% hospitalized severe cases (Programe National de Lutte contre le Paludisme [PNLP], 2019). To combat the disease, the country has developed and implemented several interventions based on prevention, care, and support strategies in accordance with the World Health Organization guidelines (World Health Organization [WHO], 2015; Programme National de Lutte contre le Paludisme [PNLP], 2018). However, malaria epidemiology is poorly understood in Togo, with a paucity of information on species of *Plasmodium* that are responsible for disease transmission. Studies on disease burden erroneously made believe that malaria cases are only related to *Plasmodium falciparum* (Pf) infection in Togo (Bakai et al., 2020; Thomas et al., 2020), making the standards of care unilateral with important implications in disease epidemiology.

Routine diagnosis using the standard method for the detection of *Plasmodium* species by examining Giemsa-stained blood smears under a microscope is the major approach being used in malaria control programmes of many tropical African settings. The method is effective and inexpensive, but it is laborious and time consuming. In addition, its sensitivity is doubtful with low parasitemia during low-transmission periods and also with the shortage of experienced technicians (Agabani et al., 1994). Polymerase chain reaction (PCR) is one of the highly sensitive methods, which has been widely used for molecular detection and identification of malaria parasites. Its effectiveness is unquestionable as it had been successfully used to detect cases of mixed infections or low parasitemia (Snounou et al., 1993c; Zhou et al., 2014). A range of PCR-based assays has been described for malaria detection including species of *Plasmodium* that regularly infect humans [Pf, *Plasmodium vivax* (Pv), *Plasmodium ovale* (Po), *Plasmodium malariae* (Pm), and *Plasmodium knowlesi* (Pk)] (Snounou et al., 1993a,b; Singh et al., 2004; Chen et al., 2017). In addition, nested PCR is used for the amplification of the small subunit ribosomal RNA (ssrRNA) gene of *Plasmodium* species (Snounou and Singh, 2002).

In order to advance knowledge of diagnosis efficacy and species of malaria parasites in Togo, this study carried out a molecular investigation on malaria clinical samples in comparison with light microscopic examination and assessed the phylogeny of *Plasmodium* species from southern Togo.

## Materials and Methods

### Study Sites

**FIGURE 1:**
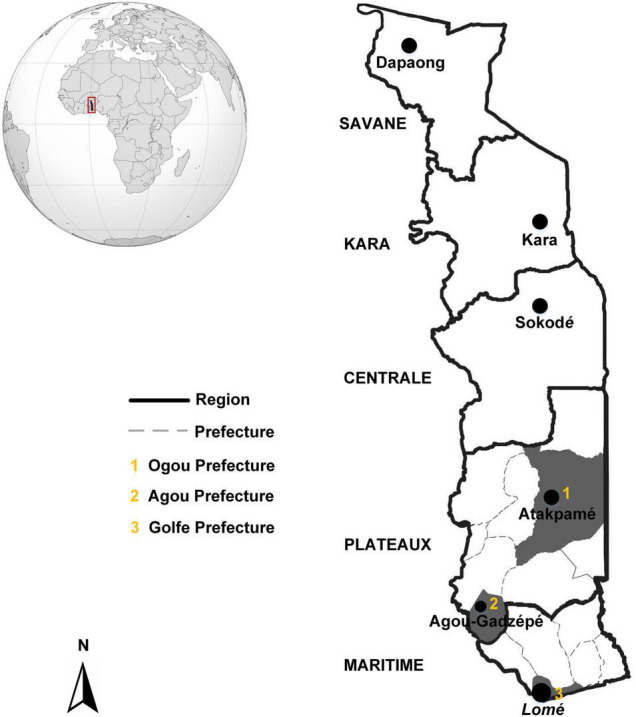
Togo, showing the geographic area of the municipalities involved in this study (dark gray shading). Atakpamé (Ogou Prefecture), Agou-Gadzépé (Agou Prefecture), and Lomé (Golfe Prefecture) are located in the Plateaux and Maritime Regions, respectively.

This study was conducted in the two southern regions (Maritime and Plateaux) of Togo (Togo Confidentiel, 2021) where malaria is endemic (World Health Organization [WHO], 2018a). Urban and periurban areas of Lomé (N 6°12′56″; E 1°22′54″) in Golfe Prefecture (Maritime Region), Atakpamé (N 7°52′87″; E 1°13′05″) in Ogou Prefecture and Agou-Gadzépé (N 7°28′01″; E 1°55′01″) in Agou Prefecture (Plateaux Region) were selected (Figure 1).

Lomé is the capital of Togo and lies on the Gulf of Guinea (Atlantic coast) in the extreme southwestern corner of the country. Its urban population was estimated at 837,437 inhabitants as of the 2010 census (Direction Générale de la Statistique et de la Compatibilité Nationale, 2010). Lomé has a tropical savanna climate despite its latitude close to the equator, which is characterized by one dry season (November–March) and two rainy seasons (April–October), with two rainfall peaks in June and September (Encyclopædia Britannica, Inc., 2021). Lomé is relatively dry, with an annual average rainfall of 800–900 mm and a mean temperature above 27.5°C (Encyclopædia Britannica, Inc., 2021). Agou-Gadzépé and Atakpamé (the largest city in Plateaux Region) are located at 80 and 161 km, respectively, from Lomé, with estimated populations of 11,000 and 84,979, respectively, as indicated in the 2006 population census (Encyclopædia Britannica, Inc., 2021). The climate in Plateaux is generally tropical characterized by dry and rainy seasons (as described above), with peaks of malaria transmission related to rainfall. The western part of Plateaux receives the highest amount of precipitation—about 1,800 mm annually (up to 2,000 mm in Agou) and, the rainfall amounts to 1,300–1,400 mm per year in Atakpamé, with annual average temperatures of 23.5 and 26°C, respectively (Encyclopædia Britannica, Inc., 2021).

### Ethics Statement

Ethical approval for malaria samples collection was assented by the Togolese Ministry of Health’s Bioethics Committee following the institutional ethical guidelines by the Ethics Committee at the National Institute of Parasitic Diseases, Chinese Center for Disease Control and Prevention, as reported previously (Kassegne et al., 2020). Informed consent was sought from all participants or their parents or guardians. Information obtained from all the participants were kept confidential.

### Patient Samples and Microscopic Examination

Lomé, Agou-Gadzépé, and Atakpamé inhabitants usually access regional hospitals, polyclinics, and community health centers experienced in the management of malaria cases for treatment and medical attention. Blood samples were collected from *Centre Médico-Social de Doumasséssé* and *Clinique Chirurgicale Martin Luther-King*, Lomé; *Centre Médico-Social Notre Dame de la Consolation d’Agbonou Campement*, Atakpamé; and *Hôpital Bethesda d’Agou Nyogbo* and *Centre Hospitalier Préfectoral d’Agou Gare*, Agou in September 2017 to December 2019. Individuals diagnosed by microscopic examination were referred to our study and samples were collected from those who consented to the study. The end of specimen collection was not determined by any factor other than the defined period of the study.

Sample collection involved febrile and suspected malaria cases including asymptomatic individuals, aged between 3 months and 70 years old. Clinical malaria patients were subjects who presented themselves to health facilities with fever or a history of fever (oral ≥ 37°C, rectal ≥ 38°C) in the last 24 h and were confirmed for Pf infection by microscopy (parasite density ranging from 2,500 to 1,000,000 asexual parasites/μl). Suspected malaria cases were persons who complained of fever of less than 2 days or having a history of fever, diagnosed either negative or positive by microscopy, and received treatment with antimalarial drugs.

Finger prick/venipuncture blood samples were obtained from individuals who received parasitological diagnosis at the health facilities, using the Giemsa-stained thick blood smear microscopic examination under × 1,000 magnification. Asexual stages of malaria parasites were counted per 200 leukocytes and, parasite density was calculated as the number of parasites per microliter by assuming a fixed leukocyte count of 8,000 cells/μl of blood. The thick film was considered positive when malaria parasites were present and negative if no parasites were seen after 500 leukocytes were counted. A total of 810 blood samples were collected including 610 Pf confirmed cases and 199 microscopy-negative cases. Except one Pm case that was detected by microscopy, no infection case of other *Plasmodium* species or case of coinfection of *Plasmodium* species was recorded from microscopy. All participants that were positive for malaria after diagnosis by parasitological blood smear test were treated with antimalarial drugs according to the national malaria programme guidelines.

### Blood Sampling and DNA Extraction

Fresh whole blood and serum samples were collected and sampled as dried blood spots (DBSs) and dried serum spots (DSSs) on Whatman FTA and 903 Protein Saving Cards (GE Healthcare, NJ, United States), respectively, according to the instructions of the manufacturer. They were then packed in transparent zip-lock airtight plastic bags with silica gel desiccant to ensure quality of spots storage. Genomic DNA was extracted from the blood samples applied to FTA Cards using the QIAGEN DNeasy Blood and Tissue Kit (Qiagen, Germany) according to the instructions of the manufacturer and as reported by Chen et al. (2017) and Shen et al. (2018). Approximately 100 μl of blood from 12 punched-out circles of 3-mm (1/8 inch) diameter each were used per filter spot. Negative controls were also included to ensure lack of contamination. A final volume of 100-μl DNA template was eluted after extraction, followed by concentration measurement using a NanoDrop ND-2000 Spectrophotometer (Thermo Fisher Scientific, NH, United States) for downstream processing.

### Polymerase Chain Reaction Amplification

T100 Thermal Cycler (BioRad, CA, United States) was used to perform nested PCR amplification of *Plasmodium* species ssrRNA genes with primers as reported previously (Zhou et al., 2014). For first-round nested PCR amplification, 2 μl of extracted genomic DNA template was added to a 25-μl PCR mixture, consisting of 0.4 M of each genus-specific primer (rPLU1 and rPLU5), 12.5 μl of 2 × Taq PCR MasterMix (Tiangen, China), which contains 0.1 U of Taq polymerase/μl, 500 μM deoxynucleotide triphosphates (dNTP), 3 mM MgCl_2_, 100 mM KCl, 20 mM Tris-HCl, and pH 8.3. Amplification of DNA targets was carried out under conditions as follows: 94°C for 5 min, 35 cycles of (94°C for 30 s, 58°C for 30 s, and 72°C for 2 min), followed by a final extension at 72°C for 10 min. Two microliters of the first PCR product were used for the second-round amplification, with conditions and concentrations identical to those used for the first, except that rPLU3 and rPLU4 were used as inner primers (Table 1) and amplification was performed at 40 cycles. The size of the amplicons using outer and inner primers during the first and nested PCRs are about 1,600–1,700 and 235 bp, respectively. These primers are genus specific and therefore, can amplify target sequences of the five species of *Plasmodium* parasites that regularly infect humans (Table 1). The first nested PCR products were used as templates for the next species-specific nested PCR amplification under the same conditions. Negative control reaction was performed in each amplification reaction. In addition, positive controls consisting of the 3D7 strain of Pf and Pv isolates from the China–Myanmar border (Yunnan Province, China), which were preserved in our laboratory, were also used.

**TABLE 1:** Primer sequences used in this study for PCR detection of malaria parasites.

PCR reaction—*Plasmodium* spp.	Primers	Primer sequences (5′–3′)	Amplicon size (bp)
Nested I—Genus specific	rPLU1	TCAAAGATTAAGCCATGCAAGTGA	1,600–1,700
	rPLU5	CCTGTTGTTGCCTTAAACTTC	
Nested II—Genus specific	rPLU3	TTTTTATAAGGATAACTACGGAAAAGCTGT	235
	rPLU4	TACCCGTCATAGCCATGTTAGGCCAATACC	
Nested II—*P. vivax*	rVIV1	CGCTTCTAGCTTAATCCACATAACTGATAC	121
	rVIV2	ACTTCCAAGCCGAAGCAAAGAAAGTCCTTA	
Nested II—*P. falciparum*	rFAL1	TTAAACTGGTTTGGGAAAACCAAATATATT	206
	rFAL2	ACACAATGAACTCAATCATGACTACCCGTC	
Nested II—*P. ovale*	rOVA1	ATCTCTTTTGCTATTTTTTAGTATTGGAGA	226
	rPLU2	ATCTAAGAATTTCACCTCTGACATCTG	
Nested II—*P. malariae*	rMAL1	ATAACATAGTTGTACGTTAAGAATAACCCC	145
	rMAL2	AAAATTCCCATGCATAAAAATTATACAAA	
Nested II—*P. knowlesi*	Pmk8	GTTAGCGAGAGCCACAAAAAAGCGAAT	153
	Pmkr9	ACTCAAAGTAACAAAATCTTCCGTA	

*bp, base pairs; PCR, polymerase chain reaction.*

### Sequencing and Analysis of DNA Targets

Two percent agarose gel electrophoresis of the PCR products was performed followed by GoldView staining and visualized under UV light using Molecular Imager Gel Doc XR^+^ Imaging System (BioRad, CA, United States). All positive results of *Plasmodium-*infected cases were confirmed again through direct sequencing of the second round nested-PCR products by the BGI Company (China), using nested primers. Sequencing reads were imported into the EditSeq module of Lasergene-Ver7.1^1^ to construct ssrRNA gene sequences of the nested-PCR products, which were then blasted at https://blast.ncbi.nlm.nih.gov/. Partial sequences of *18S ribosomal RNA* genes of the Togo isolates were aligned with those of other *Plasmodium* isolates obtained from blast analysis, using the ClustalW method (EMBL-EBI, Hixton and Cambridge, United Kingdom) of MEGA-Ver7.0^2^ to generate a phylogenetic tree based on the neighbor-joining method (Uwase et al., 2020).

### Analysis of Sensitivity, Specificity, and Predictive Values

Sensitivity and specificity are characteristics of a diagnostic test. Sensitivity is the probability that a diagnostic test indicates “disease” among those with the disease. Specificity is the fraction of those without disease who have a negative test result. Thus, sensitivity and specificity analyses for the tested population were performed as follows:

$$\text{Sensitivity}=\frac{\mathrm{True}\,\mathrm{Positive}}{\mathrm{True}\,\mathrm{Positive}+\mathrm{False}\,\mathrm{Negative}}\times 100$$

$$\text{Specificity}=\frac{\mathrm{True}\,\mathrm{Negative}}{\mathrm{True}\,\mathrm{Negative}+\mathrm{False}\,\mathrm{Positive}}\times 100$$

In addition, from a clinical perspective, in order to estimate the chance that a person with a positive diagnostic test truly has the disease or the probability that a person with a negative test does not have the disease, positive and negative predictive values (PPV and NPV, respectively) were also calculated for the population that was tested in this study.

Positive⁢Predictive⁢Value=

$$\frac{\mathrm{True}\,\mathrm{Positive}}{\mathrm{True}\,\mathrm{Positive}+\mathrm{False}\,\mathrm{Positive}}\times 100$$

Negative⁢Predictive⁢Value=

$$\frac{\mathrm{True}\,\mathrm{Negative}}{\mathrm{True}\,\mathrm{Negative}+\mathrm{False}\,\mathrm{Negative}}\times 100$$

## Results

A total of 810 blood specimens were collected in this study. Following the diagnosis results (hereinafter referred to as test results) with Giemsa-stained thick blood smear microscopic examination, we obtained 611 positive malaria cases [referred to as microscopy-positive group (MPG)] including 610 cases of Pf and one case of Pm, while the remaining 199 cases were tested malaria negative and were referred to as the microscopy-negative group (MNG). The use of *Plasmodium* genus-specific nested PCR identified 565 malaria-positive cases including 536 and 29 cases from the MPG and MNG groups, respectively. In other words, nested PCR confirmed 536 out of 611 (87%) cases that were true positive from the MPG and identified 29/199 (14.6%) malaria cases that were found negative (Table 2) in the MNG. Such a finding reveals that there were 75/245 false-positive cases (30.6% error in species diagnosis) in the MPG and 29/565 (5.1%) undiagnosed cases in the MNG. This was reflected in the sensitivity and specificity values obtained (94.9 and 69.4%, respectively), suggesting that the test missed 5% of the people who had malaria and 31% of the people tested positive for the disease which they did not have. Such errors in microscopic test might be related to inadequacy of precision in parasite-based diagnosis by microscopy or low parasitemia conditions. In conjunction, PPV and NPV for the population tested in this study were 87.7 and 85.4%, respectively (Table 2). Clinically speaking, this implies that among the people who tested positive, the chance that a person with a positive diagnostic test truly has the disease was 88% and among those who tested negative, the probability that a person with a negative test does not has the disease was 85%.

**TABLE 2:** Sensitivity, specificity, and predictive values of diagnosis for the population tested in this study.

	Test result (microscopic test)	True condition (PCR test)	
	Total (number)	Diseases (number)	Non-Diseases (number)
Positive (number)	T_Test Positive_ (611)	True Positive (536)	False Positive (75)
Negative (number)	T_Test Negative_ (199)	False Negative (29)	True Negative (170)
	Total (810)	T_Disease_ (565)	T_Non–Disease_ (245)
Sensitivity (94.9%*), Specificity (69.4%*), PPV (87.7%), NPV: 85.4%			

*T, total.*

*Sensitivity and specificity percentages of the diagnostic test compared with PCR.

**FIGURE 2:**
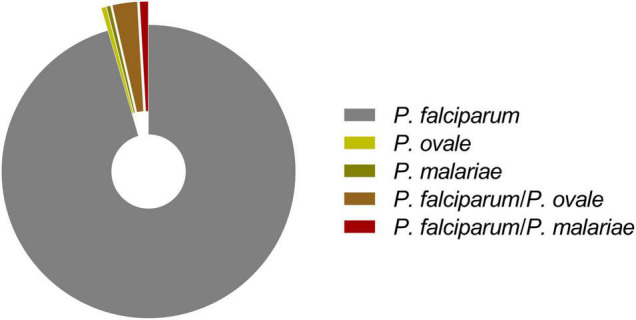
Distribution of *Plasmodium* species identified in 565 malaria infections from high-transmission areas in southern Togo, 2017–2019.

Further species-specific nested PCR and species confirmation through direct sequencing of the nested-PCR products identified higher infection rate [95.5% (540/565)] for Pf than those for other species, which were 0.5% (3/565) for Po and 0.4% (2/565) for Pm. In addition, 20 cases were coinfection cases of Pf and Po [2.7% (15/565)] and Pf and Pm [0.9% (5/565)] (Table 3). However, there was no malaria case of Pv or Pk found in this study (Figure 2).

**TABLE 3:** *Plasmodium* species identified in 565 malaria patients from high transmission areas in southern Togo, 2017–2019.

	Microscopy positive		Microscopy negative	
	*P. falciparum*	*P. malariae*		
	610	1	199	
**PCR-based species specific**	**536**		**29**	**T_number_ (%)**
	
*P. falciparum*	514	0	26	540 (95.5)
*P. ovale*	0	0	3	3 (0.5)
*P. malariae*	1	1	0	2 (0.4)
*P. falciparum*/*P. ovale*	15	0	0	15 (2.7)
*P. falciparum*/*P. malariae*	5	0	0	5 (0.9)

*T, total.*

**FIGURE 3:**
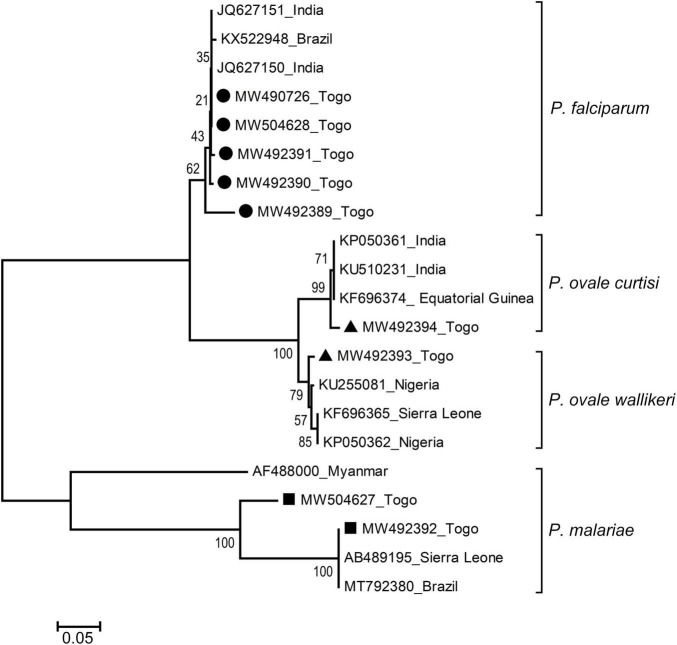
Genetic and geographical relationships of *Plasmodium* species from high-transmission areas in southern Togo based on the *18S rRNA* locus. The relationships were inferred by a neighbor-joining analysis of *18S SSu rRNA* partial gene sequences of *Plasmodium* species isolates from different countries. The numbers on the branches are percent bootstrapping values from 1,000 replicates. Each sequence of *Plasmodium* species is identified by its accession number and geographical location (country). Gene sequences of the present study are highlighted (black circles for Pf, black triangles for Po, and black rectangles for Pm). Scale bar indicates nucleotide substitutions per site.

Phylogenetic relationships of unique sequences that were amplified were constructed on the basis of similarities with published homologous sequences from different countries based on neighbor-joining analysis of the *18S rRNA* locus. Phylogenetic analysis of the fragments of *18S SSu rRNA* gene sequences of the sample cases tested in this study showed species specificity and clustered with homologous sequences of isolates of *Plasmodium* species from different countries (Figure 3). Gene sequences of malaria samples that were used for the phylogenetic analysis were deposited in the National Center for Biotechnology Information (NCBI), under GenBank accession numbers [MW490726, MW492389, MW492390, MW492391, and MW504628 (Pf); MW492393 and MW492394 (Po); MW492392 and MW504627 (Pm)].

## Discussion

Togo, an integral part of tropical Africa where malaria is endemic, has its entire population at risk of malaria infection and disease transmission occurs all year round (World Health Organization [WHO], 2018a; Kassegne et al., 2020, 2021). Sustainable efforts have been engaged to reduce malaria burden in Togo; however, effective disease control toward elimination requires novel and innovative approaches among which diagnostics, monitoring, and surveillance are of capital importance (Chen et al., 2012; Kassegne et al., 2016, 2019).

Microscopic analysis is currently being used as the appropriate test for routine clinical diagnosis of malaria, especially in low-income settings. However, the sensitivity of the detection of malaria parasites by microscopy is approximately 50–200 parasites/μl of blood (Mbanefo and Kumar, 2020). Molecular tests are very sensitive to detect *Plasmodium* species, and a number of studies demonstrated that PCR is more reliable than microscopy in detecting malaria parasites in infected individuals, especially in those with low parasite density (Roper et al., 1996; Singh et al., 1996; Zhou et al., 2014). While the population tested in this study only focused on the southern Togo, the findings revealed disease prevalence (69.8%) higher than that reported (25.5–55.1%) for the entire country (World Health Organization [WHO], 2018a). Microscopic examination had 94.9% sensitivity and 69.4% specificity, showing that the test missed 120 of the people who had malaria, and one-third of the people tested positive for the disease, which they did not have, respectively. Similar observations of misdiagnosed malaria cases by microscopy have also been reported from a molecular survey of febrile cases in malaria-endemic areas along the China–Myanmar border (Zhou et al., 2014). In this study, the test showed sensitivity aligning onto that generally estimated (95%), whereas its specificity was relatively lower (69.4%) than expected (98%) as reviewed in a recent study (Mbanefo and Kumar, 2020). That is to say, the microscopic diagnosis for this study population tested people false positive for the disease they did not have. These misdiagnoses could be attributed to lack of precision and expertise in identifying species of malaria parasites, low parasite density conditions, or inadequate staining/poorly maintained microscopes. Such an interpretation is consistent with the fact that the subset of microscopy-positive PCR-negative specimens were recorded for low parasitemia from individuals who have been under antimalarial medication. Since 2005, the Togo National Malaria Control Programme has recommended two different formulations of artemisinin-based combination therapy (ACT), artesunate–amodiaquine (ASAQ) and artemether-lumefantrine (AL), for the treatment of uncomplicated malaria as well as for the treatment of unconfirmed malaria (World Health Organization [WHO], 2018a). In other words, the same care is given irrespective of whether a falciparum or unconfirmed malaria diagnosis is made. In addition, given that routine microscopic diagnosis usually does fall short in identifying other species of *Plasmodium* than Pf, no specific drugs effective against ovale relapse or malariae infection have been reported being used in Togo. Thus, the potential misdiagnosis of malaria parasite species by microscopy does not affect current treatment standards but, instead, has important implications in malaria epidemiology because it would identify individuals without disease but for whom the test indicates “disease” or individuals who test positive for a disease which they do not have. This may prompt resistance to artemisinin or delay in parasite clearance as patients would have been given inappropriate treatment to their ailments. Another public health consequence would be that untreated patients could become carriers for malaria transmission in areas where disease diagnosis may be already “problematic”.

PCR-based molecular methods are able to detect malaria cases that are “truly positive” as well as those with mixed infections of *Plasmodium* parasites (Snounou et al., 1993b; Zhou et al., 2014). In this study, nested PCR was very specific and helped detect different monospecies of malaria cases or cases of mixed infections that were not diagnosed by microscopy. Among the five known species of *Plasmodium* that infect humans, Pf infection (and to a lesser extent Po and Pm) is the essential cause of malaria burden in tropical Africa (World Health Organization [WHO], 2018b). In West Africa, both Po and Pm malaria cases have been identified in Benin, Ghana, and Senegal (Browne et al., 2000; Roucher et al., 2014; Trape et al., 2014; Doritchamou et al., 2018), as well as Po in Cote d’Ivoire and Comoros Islands (Bauffe et al., 2012), to mention a few. However, there have been no information publicly reported yet on Po and Pm from Togo. In this study, Pf was the most prevalent *Plasmodium* species detected (95.5%) among the sample cases, aligning onto the evidence that Pf is always highly endemic in West African countries because the region usually experiences long periods of rainy reason which enhances perennial or semiperennial *Anopheles* breeding sites. We, also detected low infection rates of both ovale and malariae cases (0.5 and 0.4%, respectively). The detection of these two “bashful” malaria parasites in such a Pf-endemic area is consistent with previous studies from West Africa, which reported that Po and Pm are often associated with Pf infections, especially in areas where falciparum is highly endemic (Mueller et al., 2007; Roucher et al., 2014). However, the Po and Pm infection rates found in this study were much lower than those reported from studies in other West African countries [e.g., 15.5% Po and 10.4% Pm infections in a community-based survey in Ashanti Region of Ghana (Browne et al., 2000); 9.2% Pm and 5.8% Po infections in a cohort in Beninese pregnant women (Doritchamou et al., 2018), or 2.5% Po and 12.2% Pm infections in a longitudinal study in Dielmo Village, Senegal (Roucher et al., 2014)]. The large-scale cohorts conducted over the years in such malarious areas might explain the high infection rates observed for both Po and Pm. Furthermore, there was evidence that Po and Pm are sympatric with Pf across the African continent and are frequently present as coinfections (Fancony et al., 2012). Low-level infection rate with Po or Pm seems to be common across tropical Africa in areas where malaria is endemic and often as complex mixed infections with Pf. The rate of Pf coinfections with Po or Pm was reported to be about 24% in the rainforest area of Ghana (Browne et al., 2000), while the proportion was much lower in other parts of West Africa such as Dielmo Village, Senegal (7.9% Pf/Pm and 1.1% Pf/Po mixed infections) (Roucher et al., 2014) or among Beninese pregnant women (6.6% Pf/Pm and 2.3% Pf/Po mixed infections) (Doritchamou et al., 2018). The low mixed infection rates that we observed in this study may be related to that all the samples were mainly collected in urban areas. However, we found infection rates of coinfection cases for both Po and Pm with Pf higher than the rates for single Po or Pm infection cases. This was consistent with previous findings from Ghana where it has been found that cases infected with Pf are more likely to carry Po or Pm than those who were not infected with Pf (Browne et al., 2000). Such a finding suggests evidence for interactions between Pm or possibly Po, with Pf infections (Mueller et al., 2007).

A series of comprehensive studies have investigated Pf in different parts of tropical Africa where malaria is endemic. However, morbidity associated with Po and Pm infections has received relatively little attention and was, therefore, markedly underestimated and unreported because they are generally regarded as a benign cause of malaria. Such interpretations could be attributed to the following reasons: (i) low incidence of the disease, (ii) lack or rarity of severe cases as they are regarded less important to Pf in terms of public health issues, or (iii) practical difficulties in microscopic identification as Po and Pm share morphological characteristics resembling those of Pf (young ring forms of the three species may be difficult to distinguish in thick blood films, especially during coinfections) (Roucher et al., 2014). Thus, in malaria-endemic areas of tropical Africa including Togo, most of all malaria attacks are erroneously attributed to Pf, probably due to underdiagnoses of Po and Pm clinical attacks. This study showed evidence of Po and Pm coexistence with Pf in southern Togo. More so, ovale or malariae malaria would have already been diagnosed by microscopy as falciparum malaria due to similarities in symptoms and species morphology. PCR-based diagnostic tests of clinical cases are therefore required for accurate species discrimination and detection.

Although considered “mild,” Po and Pm may constitute an important cause of morbidity and high risk of mortality. For example, fever episodes related to parasitemia peaks of Pm (in Liberia and The Gambia) or Po (in Senegal), in both children and adults have been reported (Miller, 1958; Greenwood et al., 1987; Faye et al., 2002). Also, Pm can cause chronic infections that can persist in the host for many years and reoccur after living in endemic areas (Siala et al., 2005); e.g., a chronic nephrotic syndrome that, once established, does not respond to treatment and carries a high rate of mortality (Mueller et al., 2007). Po shares peculiarity with Pv to form the latent stage in the liver—hypnozoite, which causes late relapse of the parasite, with new febrile episodes (after months or years) without recent exposures (Coldren et al., 2007). The reinforcement of malaria control policies for accurate diagnosis of parasites species in Togo should be, therefore, prioritized, to ensure deployment of appropriate treatment regimen for malaria patients and to provide a better knowledge on epidemiological assessments to guide control interventions.

## Conclusion

This study publicly reports, for the first time, a molecular survey of malaria infections in Togo and also reveals the presence of other species of malaria parasites—Po and Pm, which were not previously reported. In addition, findings from this study indicated errors in microscopic examination including error in species diagnosis and undiagnosed cases. For example, in this study population, one-third of the people were microscopically tested positive for the disease they did not have. Thus, overlaps of different species of malaria could further aggravate the disease burden in Togo if appropriate actions are not taken, especially in situations where diagnosis of monospecies of malaria cases seems already “problematic.” Collectively, this preliminary study advanced our understanding of the malaria epidemiology in southern Togo and provided information to improve disease control/surveillance policy. However, large-scale studies across the whole country are required in the future to assess malariae and ovale malaria epidemiology.

## Data Availability Statement

The datasets presented in this study can be found in online repositories. The names of the repository/repositories and accession number(s) can be found below: https://www.ncbi.nlm.nih.gov/, MW490726, MW492389, MW492390, MW492391, MW504628, MW492393, MW492394, MW492392, and MW504627.

## Ethics Statement

Permission was obtained from all malaria subjects before collecting the specimens. Blood collection was made under a study protocol reviewed and approved by the Togo Ministry of Health’s Bioethics Committee (Authorization N°019/2019/MSHP/CBRS), following institutional ethical guidelines by the Ethics Committee at the National Institute of Parasitic Diseases, Chinese Center for Disease Control and Prevention, as reported previously (Kassegne et al., 2020).

## Author Contributions

KK and J-HC conceptualized the study. KK, KA, KN, and KKK collected and analyzed the specimens. KK, S-WF, and KKK conducted the experiments. KK interpreted the data and wrote the manuscript. EMA, X-KG, and X-NZ revised the manuscript critically for intellectual content. All authors contributed to the article and approved the submitted version.

## Conflict of Interest

The authors declare that the research was conducted in the absence of any commercial or financial relationships that could be construed as a potential conflict of interest.

## Publisher’s Note

All claims expressed in this article are solely those of the authors and do not necessarily represent those of their affiliated organizations, or those of the publisher, the editors and the reviewers. Any product that may be evaluated in this article, or claim that may be made by its manufacturer, is not guaranteed or endorsed by the publisher.

## Footnotes

1
https://lasergene.software.informer.com/7.1

2
http://mega.software.informer.com/7.0/
